# Barley roots require *HvGLU3* for cellulose biosynthesis

**DOI:** 10.1093/plphys/kiaf326

**Published:** 2025-07-23

**Authors:** Blanca Jazmin Reyes-Hernández

**Affiliations:** Assistant Features Editor, Plant Physiology, American Society of Plant Biologists; Section for Plant Glycobiology, Department of Plant and Environmental Sciences, Faculty of Science, University of Copenhagen, Frederiksberg C 1871, Denmark

Plant cells are enclosed by the cell wall, a complex polysaccharide structure that strengthens the plant body and supports plant development, cell communication, and immunity. The cell wall is composed of different layers. The outermost layer, the middle lamella, is shared between adjacent cells and holds them together. Between the middle lamella and the plasma membrane lies the primary cell wall. In some specialized cells that require greater mechanical strength, a secondary cell wall is developed inside the primary wall. Primary and secondary walls consist mainly of cellulose and hemicelluloses; the primary wall also contains pectin while the secondary is enriched in lignin (Cosgrove 2005).

Cellulose is synthesized at the plasma membrane by cellulose synthase complexes (CSCs). The resulting fibrils contain very ordered tough crystalline regions and looser amorphous parts, giving the cell wall its strength and some flexibility (Cosgrove 2005).

In Arabidopsis, the membrane-anchored endo-1,4-ß-D-glucanase KORRIGAN1(KOR1) is a key component of CSCs (Mansoori et al. 2014). Endo-1,4-ß-D-glucanases are enzymes that break down long chains of polysaccharides such as cellulose that are joined by a 1,4-ß-D glucan bond (Perrot et al. 2022). KOR1 is suggested to trim nascent 1,4-β-D glucan chains rather than break down mature tough crystalline cellulose (Mølhøj et al. 2001). Its correct delivery from the endoplasmic reticulum through the Golgi to the membrane is tightly regulated. When KOR1 is absent or mislocalized, cellulose fibers are poorly assembled, and the new nascent cell walls (cell plate) fails to close completely during cytokinesis, an effect that is more severe under salt stress (Nagashima et al. 2020). Despite the importance of KOR1 in cellulose biosynthesis and cell division, the mechanism by which endo-1,4-ß-D-glucanases regulate these processes remains unexplored.

In a recent study published in *Plant Physiology* (Guo et al. 2025), a barley mutant with short roots caused by repressed cell division and elongation was identified. This mutant, which originated from chemical mutagenesis, displayed twisted roots with a rough surface resembling roots with problems in cellulose biosynthesis. Gene mapping showed that the affected gene was *HvGLU3*, which encodes for a putative ß-endoglucanase. This mutant was designated as *hvglu3-1* and presents a single-nucleotide substitution causing an amino acid change from serine to asparagine residue at position 178 of the protein.

A second allele was identified with a premature stop codon and a more severe phenotype, confirming that the mutant phenotype was caused by the loss of HvGLU3 function. Although the aerial parts of the *hvglu3-1* mutant look normal, they are smaller than the wild type. Cross sections in the root of the mutant indicated that, despite an overall normal pattern, (i) the epidermis was thicker in the meristem region and (ii) the cells of this tissue were lost in the elongation zone above. Reverse transcription quantitative PCR experiments demonstrated that *HvGLU3* expression was normally high in the elongation and meristematic zones, while it was lower in shoot and leaf, supporting the idea that its role could be relevant in the root (Fig.).

**Figure.**
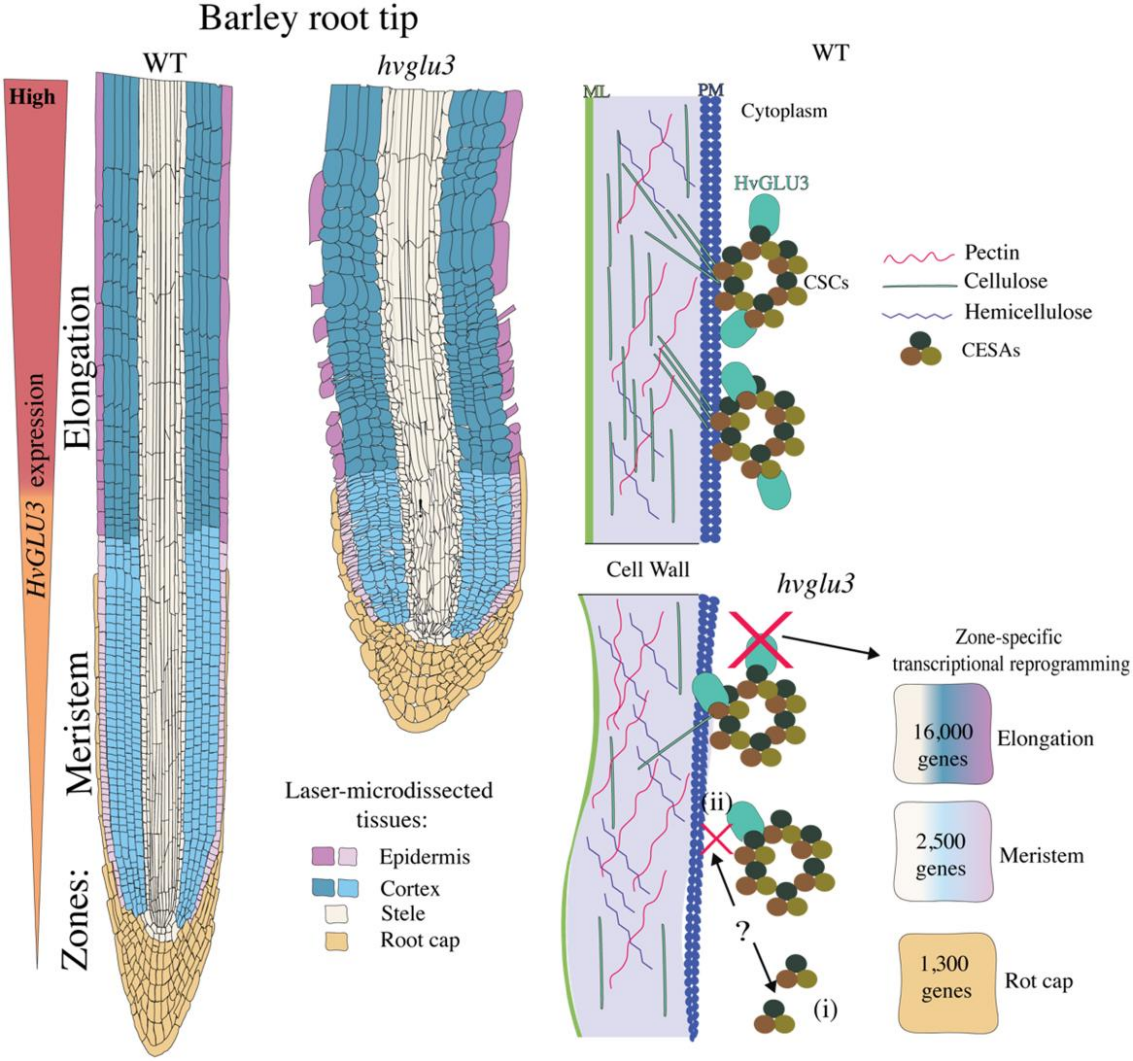
*HvGLU3* is essential for root development and cell wall integrity in barley. This schematic representation of barley wild type (WT) and *hvglu3-1* mutant root tips is based on the findings by Guo and collaborators (2025). On the left, WT root shows stronger *HvGLU3* expression in the elongation zone than in the meristem. The mutant root is shorter, with a reduced meristem, shorter cells, thickened epidermis in the meristem, and a loss of epidermal cells in the elongation zone. On the right, a comparison of cell wall structure shows decreased levels of cellulose and possibly higher levels of hemicellulose and pectin in *hvglu3-1*, all of which contributes to tissue- and zone-specific transcriptional changes. Laser capture was performed on the indicated tissues for transcriptional analysis. Cellular components bordering the cell wall are indicated, such as the middle lamella (ML) and plasma membrane (PM). The top-right diagram illustrates the putative interaction between HvGLU3 and CESA proteins under WT conditions, as suggested by their colocalization in tobacco. The bottom-right model proposes 2 possible ways (?) in which loss of *HvGLU3* might disrupt cell wall biosynthesis: (i) by directly impairing CSC activity or (ii) by eliminating HvGLU3's enzymatic function. Numbers indicate the approximate number of genes reprogrammed in each zone following *HvGLU3* loss of function. Overall, HvGLU3 is required for proper cellulose synthesis: it modulates the balance of structural and protective polymers in the cell wall and regulates gene expression across root zones.

Phylogenetic and sequence analyses revealed that *HvGLU3* homologs are present throughout the plant kingdom and that the gene is highly conserved within the barley germplasm*. HVGLU3* is ortholog of the rice gene *OsGLU3*, which is involved in cellulose biosynthesis (Zhang et al. 2012). Rice *OsGLU3* mutants display opposite phenotypes: some accumulate cellulose; others lose it (Inukai et al. 2012). Consistent with the cellulose-deficient root phenotypes observed in *osglu3-1*, *osglu3-2* (Zhang et al. 2012), and their Arabidopsis homolog *kor1* (Takahashi et al. 2009), *hvglu3-1* mutant also exhibits reduced cellulose content.

To investigated *HvGLU3* function in barley, the authors treated seedlings with isoxaben, a cellulose biosynthesis inhibitor. Wild type roots stopped elongating under treatment, while *hvglu3-1* roots hardly changed, meaning that *hvglu3-1* already has a cell wall defect. Biochemical assays confirmed a 60% drop in cellulose content in *hvglu3-1*. At the same time, changes in sugar composition shifted, indicating an increase in arabinoxylan hemicellulose and pectin levels, likely a compensatory mechanism to reinforce the weakened cell wall. In addition, suberin levels were reduced in *hvglu3-1*, pointing to a compromised protective barrier. Histologic analysis supported these biochemical results, altogether indicating that *HvGLU3* is required for proper normal cellulose deposition and for maintaining the correct balance of structural and protective components in the barley root cell wall.

To gain insight into the function of HvGLU3, the authors transiently expressed it in tobacco leaves using *RFP-HvGLU3* with mVenus-tagged cellulose synthases (CESAs), revealing colocalization at the plasma membrane. These interactions were confirmed by a bimolecular fluorescence complementation assay, suggesting that HvGLU3 interacts with CESAs and could be part of the cellulose biosynthesis machinery in barley.

To explore how defective cellulose biosynthesis in *hvglu3-1* affects the plant at a molecular level, the authors performed RNA sequencing on specific root tissues using laser capture microdissection. They isolated the stele, cortex, epidermis, and root cap, separating cells from the meristem and elongation zones. The elongation zone exhibited the strongest differential transcriptional response when compared with wild type roots, with >16,000 genes differentially expressed, as opposed to about 2,500 in the meristem and 1,300 in the root cap. In all tissues, upregulated genes were 2 to 3 times more abundant than downregulated ones. Genes involved in cellulose biosynthesis were mostly downregulated, while lignin-related genes were upregulated in mutant roots. These results indicate that the defect in cellulose biosynthesis in the mutant *hvglu3-1* leads to a substantial transcriptomic reprogramming across various root tissues.

To understand broader patterns of gene expression, the authors used coexpression network analysis and found 18 gene modules linked to specific tissue types. Each module included hub genes highly connected to key biological functions, such as those related to the elongation zone, meristem cortex, and root cap. A separate analysis focused on genotype differences indicated 9 modules associated with *hvglu3-1* or wild type. Some of these overlapped with tissue-specific modules, showing that gene expression changes in the mutant depend on tissue type and genotype.

This study highlights *HvGLU3* as a key endoglucanase needed for cellulose production and root development in barley. Its loss not only reconfigures the transcriptome but also leads to epidermal damage. As yet, it is not known whether HvGLU3 works by cutting glucan chains enzymatically or by helping the delivery or function of CSCs. The authors also raise new questions—for example, what signals cause the increase in lignin and drop in suberin in mutant roots? The hub genes found in the elongation zone epidermis may play a role in these shifts. Overall, these findings link cell wall building blocks to tissue development and open new possible ways to tune plant resilience.

## Data Availability

No additional data were produced or assessed to support this research.
